# Pterygoid Hamular Bursitis: A Possible Link to Craniofacial Pain

**DOI:** 10.1155/2018/5108920

**Published:** 2018-08-12

**Authors:** Sameep S. Shetty, Premalatha Shetty, Prit Kiran Shah, Jayanth Nambiar, Nancy Agarwal

**Affiliations:** ^1^Department of Oral and Maxillofacial Surgery, Manipal College of Dental Sciences, Manipal Academy of Higher Education, Mangalore 575001, India; ^2^Consultant Oral and Maxillofacial Surgeon, My Dentist and Bhatia Hospital, Mumbai, India; ^3^Department of Conservative Dentistry and Endodontics, Sharavathi Dental College, Shiomoga, India

## Abstract

A striking feature of the skull base is the pterygoid hamulus known for its bizarre morphology and biomechanical location. Pterygoid hamular bursitis is an inflammation of bursae located between the tendon, muscle, and bony prominences. The minimal objective finding in an apparently normal orofacial apparatus and dependence on the subjective symptoms experienced by the patient with widespread referral pattern often perplexes the clinician. Bursitis should be considered in the differential diagnosis of craniofacial neuralgia, temporomandibular joint dysfunction, and chronic craniofacial pain. Clinical signs and symptoms of this intriguing entity are diverse and multifaceted that can sometimes demand services of clinicians across various specialties considering the anatomic density of the region. Care must be taken to avoid delay, misdiagnosis, and overtreatment.

## 1. Introduction

Chronic orofacial pain is a complex process whose causes originate from the trigeminal nociceptive reflex arcs within the central and peripheral nervous systems. It comprises a highly extensive spectrum of pain disorders because of their unique anatomic, physiologic, and biochemical components.

Patients with persistent orofacial pain do not present with concrete symptoms that allow for a straightforward clinical reasoning. Orofacial pain can have a blend of neurogenic, myogenic, and psychogenic causes entangled amidst the intricate anatomy and high vascular network. This umbrella of uncertainty shades the judgment for every patient to whom we provide care.

The clinical course of a chronic orofacial pain often seems to worsen the quality of life with polypharmacy and doctor shopping that have their own repercussions eventually leading to overdiagnosis and overtreatment.

Pain in the soft palate due to an elongated pterygoid hamulus was first termed by Gores [1]. Hjørting-Hansen and Lous [2, 3] in 1987 designated the term “pterygoid hamulus syndrome” characterized by pain in the palatal and pharyngeal regions, caused by a bizarre shape or an elongated pterygoid hamulus. Pterygoid hamulus bursitis is one such rare entity showing a constellation of symptoms in the orofacial region. The apparently normal clinical picture of the oral cavity can perplex the clinician.

The pterygoid hamulus is a variable anatomical structure with its subdivisions: base, corpus, sulcus, and caput. The average measurements are length 7.2 mm, sagittal breadth 1.4 mm, and transverse breadth of 2.3 mm. The muscle attachment within the hamulus subjects its body to a greater load in the mediodorsal direction, while the head of the hamulus can be freely pulled away laterally and caudally. The bare position of the hamulus at the distal end of the upper dental arch and the presence of a bursa or sliding layer in the sulcus suggest that it could be a possible source for trauma or infection [4].

The symptoms of pterygoid hamulus bursitis cited by Shankland are multifaceted; pain in the hamular region, palatal pain, earache, odynophagia, maxillary pain, dysphagia, and localized erythema [5]. Some of its clinical presentations may mimic glossopharyngeal neuralgia, temporomandibular joint disorders.

Vigilance of this intriguing entity is essential to all health practitioners concerned in the diagnosis and management of orofacial pain. In patients with undiagnosed neck and/or facial sporadic pain, an elongated pterygoid hamulus or a styloid process needs to be included in the diagnostic cascade of orofacial pain. It would be worthwhile to diligently listen to our patients while they narrate their history as this can distill a plethora of maladies involving the orofacial apparatus and adopt a dual axis approach.

The purpose of this article is to present a case of bursitis that was managed by surgical shaving of pterygoid hamulus following a failure of conservative management and discuss its complex pain referral pattern.

## 2. Case Report

A 42-year-old female presented to us with a primary complaint of pain in the left side of her face for 3 yrs. The pain was spontaneous and oppressive in nature. She had a history of burning, a pricking type of dysesthesia (pins and needles feeling), intermittent in nature and radiated to the left temporal and orbital region. The unremitting nature of pain often made her feel anxious and agitated with lack of sleep. No trigger factors and aggravating or relieving factors were disclosed in the history. She narrated a history of uneventful extraction of a decayed upper third molar and a restoration of carious tooth citing as a possible source of pain by her dentist.

Her medical history was unremarkable except the ingestion of a cocktail of medicines alternating from analgesics, antibiotics, steroids, and antidepressants prescribed by multiple physicians for the unremitting chronic pain she was experiencing. An array of investigations was performed ranging from MRI brain, OPG, and cephalograms that turned out to be inconclusive. Vascular decompression, central pontine dysfunction, skull base, and metastatic tumor were ruled out following the normal slices seen in MRI and CT. Routine chair side diagnostic tests were done to rule out odontogenic pain.

**Figure 1:**
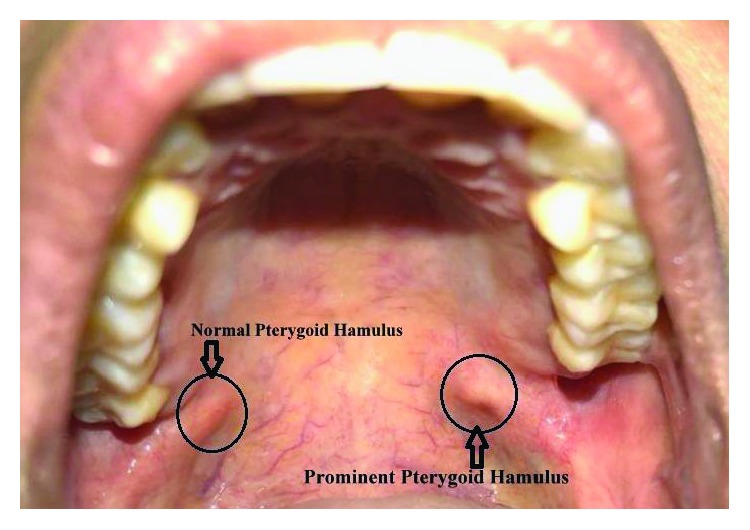
Prominent hamular process with a knife-edge bony projection on the left side.

**Figure 2:**
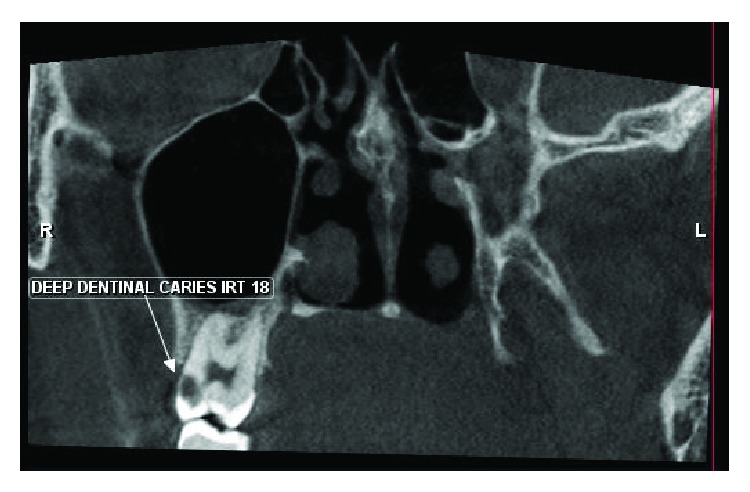
Coronal view showing the presence of dentinal caries on the contralateral side.

**Figure 3:**
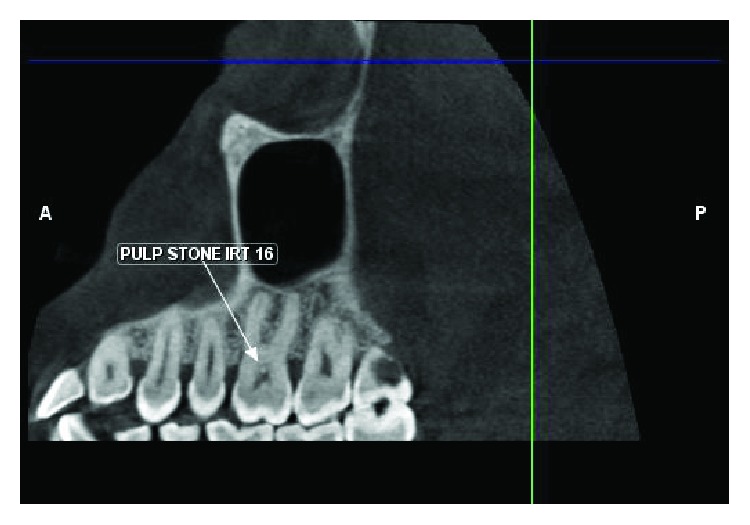
Coronal view showing the presence of pulp stones on the contralateral side.

On clinical examination, a sharp localized pain in the hamular region was evident on palpation due to the elongated hamular process that had a knife-edge bony projection (Figure 1). The overlying palatal mucosa had no change in color or texture. A local anesthetic (1 ml of 2% lidocaine) infiltration was injected with subsequent impermanent relief of symptoms in a localized area. Her oral examination was nonremarkable on the affected left side with deep dentinal caries with respect to 18 (Figure 2) and pulp stones with respect to 16 on the right side (Figure 3). Blood investigations carry less significance except in the possible diagnosis of cranial arteritis and for autoimmune disorders such as Sjogren's syndrome.

**Figure 4:**
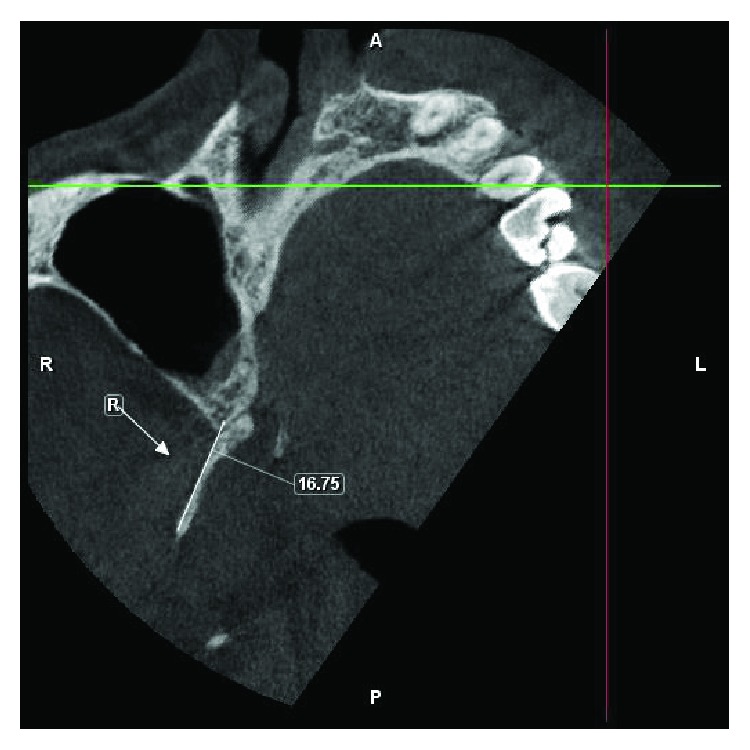
Cone beam computed tomography axial view showing an elongated pterygoid hamulus.

**Figure 5:**
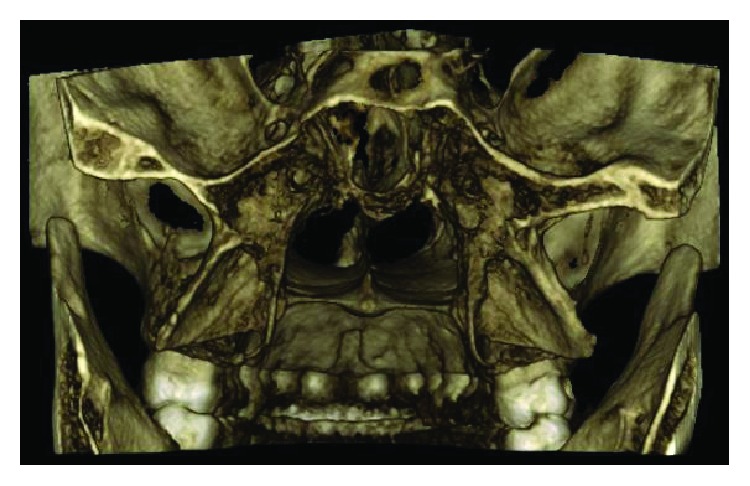
3-D reconstructed skimmed view showing the elongated pterygoid hamulus.

**Figure 6:**
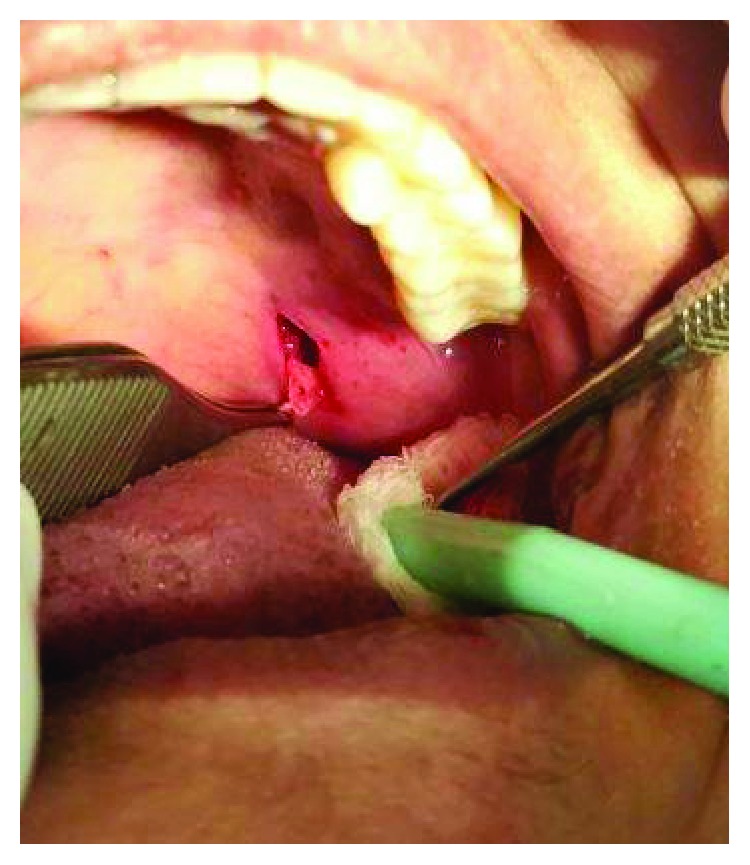
Surgical resection of the elongated and the prominent pterygoid hamulus from its base.

Following a failure of conservative remedies in the past, a prominent elongated hamular process (18.53 mm) noticed on a cone beam computed tomography: axial section (Figure 4), 3-D reconstructed view (Figure 5), and a positive diagnostic block [6], we opted for a surgical shaving in pursuit of pain relief (Figure 6).

**Figure 7:**
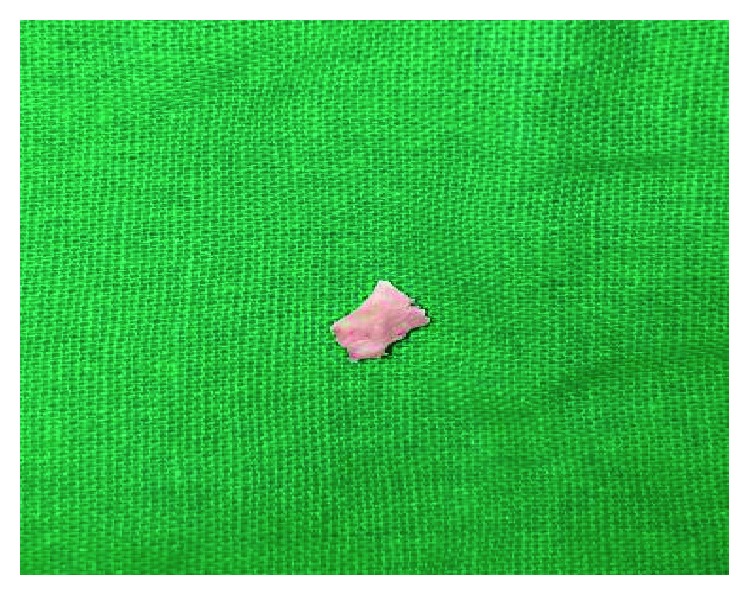
Resected hamular process resembling an arrowhead.

A longitudinal incision of the mucosa was planned along with dissection up to the pterygoid hamulus followed by resection of the hamulus from its base. The gross specimen measured 13 mm in length and its shape resembled an arrowhead (Figure 7).

## 3. Discussion

Pain in the orofacial region takes a complex route when it progresses to a chronic state. The neurologic, myogenic, and psychogenic symptoms can overlap in this intricate, innervated anatomic region. A “trigger zone” is a hallmark of both myofascial pain dysfunction and neurologic states.

The “trigger zone” in myofascial pain is a confined tender area on a firm band of skeletal muscle, tendon, or ligament. The pattern of pain referral for each trigger point is consistent. Contrary to this neurologic pain, states are categorized by voluntary or involuntary activation of painful paroxysms by tactile or thermal stimulation of certain superficial areas supplied by the afflicted nerve [7, 8].

In our case, the absence of tenderness over the muscles of mastication and the spontaneous complex pain referral pattern without any trigger factors made the diagnosis of myofascial pain dysfunction syndrome and craniofacial neuralgia a questionable one [9].

Bursae are fluid-filled compacted sacs with synovial fluid that acts as a cushion between bones, tendons, joints, and muscles. Anaesthesia intubations, swallowing a big bolus, yawning, sustained overextended maxillary prosthesis, traumatic injury while brushing, bulimic patients, and “fellatio” in child sexual abuse are cited as possible perpetuating factors for this intriguing entity [10, 11]. In our patient with no history of major surgery under general anaesthesia and any significant history of comorbidity, a possible etiological factor could be a traumatic injury. Hamular bursitis can trigger referred craniofacial pain, which may mimic a cluster of symptoms as seen in temporomandibular disorders, impacted teeth, trigeminal and glossopharyngeal neuralgia, stylohyoid ligament calcification, stylomandibular ligament inflammation, tumors, and otitis media [12].

Treatment of pterygoid hamulus bursitis per se remains ambiguous with no clear-cut consensus so far. It can be either conservative or surgical. Injection of steroids has been used with a mixed bag of results [5, 13].

The trigeminal nerve innervates anatomically related but functionally diverse anatomic structures such as the meninges, auricle, dentofacial apparatus, and eyes. A large proportion of the sensory cortex > 40% receives the trigeminal input and overlap of trigeminal sensory nucleus with the upper cervical dermatomes in the brain stem [14] seems to be a plausible explanation for the persistent pain in the orofacial region that has functional and psychological consequences.

The baffling nature of the coexisting pain conditions distant from the source of nociception had perplexed us in arriving at a definitive diagnosis. In addition, the bone of contention was “can such a small bony spicule be a source of craniofacial pain?” Although it is inviting to speculate that the intricate vascular and diverse neural networks within the tensor tympani muscle attached to the pterygoid hamulus as a plausible explanation, it cannot be substantiated by the scarcity of reports published in the literature. Sasaki et al. [15] opined that bursitis inhibits the muscular contraction of the tensor veli palatini muscle due to the mechanical stimulation by the bony spicule or osteophyte. This in turn may stimulate the branches of the greater and lesser palatine nerve, glossopharyngeal nerve, and facial nerve, which may result in widespread painful sensation following the peripheral distribution of the nerve.

**Figure 8:**
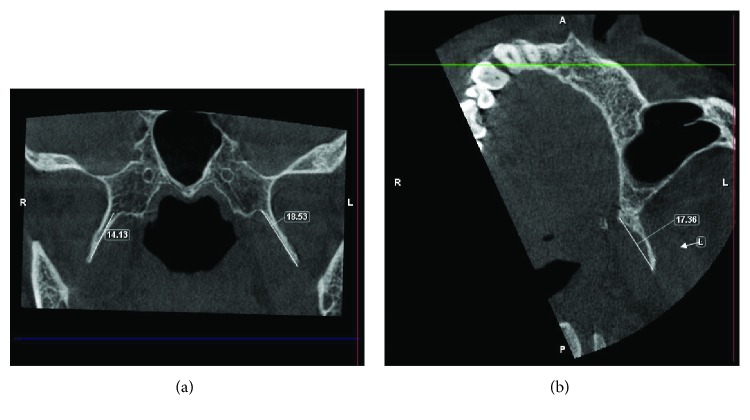
Axial view depicting an elongated pterygoid hamulus measuring about 18.53 mm on the left side and 14.13 mm on the right side.

Amongst the growing body of evidence pertaining to the position and morphology of pterygoid hamulus in different populations, the average length was found to be within the range of 4.9 to 7.2 mm [15–20]. This is in contrast to the length of 18.53 mm seen in our case (Figures 8(a) and 8(b)). The prominent bony spicule were suggestive of some mechanical stimulation to the neighbouring tissues by the elongated pterygoid hamulus eliciting pain that perhaps impaired the smooth functioning of the tensor veli palatini muscle. Confirming the elongated pterygoid hamulus radiographically, a reasonable anatomic postulation could have been existed: (1) soft palate mucosa may have been thin and friable than usual; (2) soft palate mucosa may have been much closer than usual to a rather normally located hamulus making it vulnerable to any frictional trauma [21]; and (3) asymmetry of the Pterygoid plates [22].

In addition to the surgical procedure, she also had a session of cognitive behavioral therapy considering the psychosocial aspect of chronic orofacial pain. We had maintained a pain diary that is often considered an indispensable part of assessment of treatment outcome of chronic orofacial pain in terms of pain severity, frequency, and duration of pain. The pain had substantially diminished several months post therapy; this can be attributed to the central sensitization, secondary adjacent muscle hyperactivity, hormonal fluctuations in a female gender, and neuroplasticity, a common phenomenon in chronic pain conditions [23–25]. It should be noted that as the duration of the suffering is protracted, the emotional vector of pain persists despite the inflammatory response to the acute cause of pain has ceased.

## 4. Conclusion

The superficial location of bursa and its functional orientation predisposes it to traumatic injury and chronic inflammation. A possible diagnosis of hamular bursitis should be considered, while diagnosis of chronic orofacial pain as the management for bursitis is quite inimitable.

This case illuminates the implications of a quiescent pterygoid hamulus that can perplex the clinician by causing craniofacial pain. In addition, the constellation of symptoms mimicking the other maladies of the orofacial apparatus can lead to misdiagnosis and mistreatment. A slice of wisdom allied from this case report is to listen to your patients, amalgamate the information than trivializing it, and always consider multifaceted nature of chronic orofacial pain. There has always been a constant communication between the mind and body, and chronic pain patients need to have a positive outlook. They need to align their thoughts before it malaligns them and their nervous system.
